# Exploring dietary approaches in the prevention and management of Amyotrophic Lateral Sclerosis: A literature review

**DOI:** 10.3934/Neuroscience.2023028

**Published:** 2023-11-29

**Authors:** Ubaid Ansari, Jimmy Wen, Isabel Taguinod, Dawnica Nadora, Denise Nadora, Forshing Lui

**Affiliations:** California Northstate University College of Medicine, USA

**Keywords:** amyotrophic lateral sclerosis, neurodegenerative disease, mediterranean diet, vegan diet, carnivore diet, paleolithic diet, ketogenic diet

## Abstract

Amyotrophic lateral sclerosis (ALS) is a fatal and complex neurodegenerative disease of upper and lower motor neurons of the central nervous system. The pathogenesis of this multifaceted disease is unknown. However, diet has emerged as a modifiable risk factor that has neuroprotective effects towards other neurological disorders such as Alzheimer's, Parkinson's and dementia. Thus, this review aims to explore how diet can potentially influence ALS onset and/or progression. In this review, five popular diets (Mediterranean, Vegan, Carnivore, Paleolithic and Ketogenic) and their distinct macromolecule composition, nutritional profile, biochemical pathways and their potential therapeutic effects for ALS are thoroughly examined. However, the composition of these diets varies, and the data is controversial, with conflicting studies on the effectiveness of nutrient intake of several of these diets. Although these five diets show that a higher intake of foods containing anti-inflammatory and antioxidant compounds have a positive correlation towards reducing the oxidative stress of ALS, further research is needed to directly compare the effects of these diets and the mechanisms leading to ALS and its progression.

## 1. Introduction

Amyotrophic Lateral Sclerosis (ALS), commonly known as Lou Gehrig's disease, is a devastating neurodegenerative disorder in which there is progressive degeneration of upper and lower motor neurons in the brain and spinal cord [1]. ALS is a life-changing diagnosis with no known cure. This debilitating condition leads to muscle weakness, loss of motor function and ultimately respiratory failure [1]. An emerging area of interest in the search for effective ALS treatments investigates the potential influence of dietary factors in both preventing and slowing disease progression. In recent years, the relationship between diet and neurological disorders has gathered significant attention from researchers and the public alike. The particular dietary patterns that have come under scrutiny for their potential impact on ALS are the mediterranean diet, vegan diet, carnivore diet, paleo diet and ketogenic diet. Each of these diets is characterized by its own distinct nutritional profiles. Their potential effects on neuroprotection, inflammation reduction and overall metabolic health have led to them being considered potential interventions for ALS.

The Mediterranean diet, possibly the richest in antioxidants, has been shown to reduce the oxidative stress related to neuron death seen in ALS [2]. The vegan diet's wealth of plant-based foods, such as fruits, vegetables and whole grains offers a rich supply of antioxidants and anti-inflammatory compounds, potentially reducing chronic neuroinflammation associated with the development of ALS [1]. Additionally, the absence of animal-based fats is compensated by incorporating plant-based sources of healthy fats, like avocados, flaxseeds and walnuts, which are abundant in omega-3 fatty acids, potentially providing neuroprotection and lowering the risk of ALS [3]. The carnivore diet has conflicting evidence regarding its benefit or detriment in patients with ALS. One study showed that an increased consumption of fats and proteins, especially those derived from meat during the initial phase of the illness, may extend the lifespan of individuals with ALS [4]. In a separate study, ALS was revealed to have a positive link to the intake of overall protein, glutamate and animal proteins [5]. Symptoms of ALS may be alleviated by the Paleo diet, which studies have shown encourage a higher consumption of omega-3 fatty acids, disrupting the activation of the nuclear factor kappa-light-chain-enhancer of activated B cells (NF-κB) pathway and ultimately inhibiting inflammation as pro-inflammatory cytokines will not be released [6],[7]. Finally, ketogenic diets have been shown to successfully work as a therapy to manage a variety of neurodegenerative conditions [8].

This comprehensive literature review aims to shed light on the existing body of research surrounding these diverse dietary approaches and their roles in preventing or slowing the progression of ALS. By critically examining the available scientific evidence, this review hopes to provide a nuanced understanding of how dietary choices may influence ALS onset and progression, offering potential insights into novel therapeutic strategies or lifestyle modifications for individuals at risk of or already affected by this devastating disease.

## 2. Review

### 2.1. Mediterranean Diet

The Mediterranean diet (MedD) was established in the 1960s by Ancel Keys, an American nutritionist, and is based on the eating habits of people in Greece, Southern Italy and Spain [9]. This diet mostly consists of a high intake of olive oil, vegetables, fresh fruit, dairy products such as cheese or yogurt, fish, and poultry eaten in low to moderate amounts [9]. It also includes eggs, red meat and red wine in low to moderate amounts [9]. Since MedD is characterized by nutrient-dense foods, derived from vegetables and fruits, this suggests that it may have a protective role against age-related cognitive decline and mild cognitive impairment. In a recent prospective cohort study done by Aundreu-Reinon et al., they reported that MedD was inversely associated with dementia [10]. They found that the healthy participants who adhered to the MedD had a 20% lower risk of dementia as compared to those with low adherence to MedD [10]. This may likely be due to the neuroprotective effects of the MedD since it is rich in antioxidants, fiber and omega-3 polyunsaturated fatty acids [11]. In particular, flavonoid antioxidant is protective against free radical damage by scavenging reactive oxygen species, activating antioxidant enzymes and inhibiting oxidases such as xanthine oxidase, cyclooxygenase, lipoxygenase and phosphoinositide 3-kinase, while also reducing α-tocopherol radicals [12]. As a result, this allows for certain nutritional interventions such as the Mediterranean diet rich in antioxidants, such as flavonoids, to lower the risk of neurodegenerative diseases.

Among all the existing diets that can be followed, the Mediterranean diet is possibly the richest in antioxidants. Because of this, it has been suggested that MedD can alleviate ALS symptoms despite it being an incurable neurodegenerative disease since it can ultimately reduce the oxidative stress related to neuron death seen in ALS. MedD, which is known to be rich in polyphenols, a potential amyloid aggregation inhibitor, has been reported to be able to reduce neurodegeneration because of its high olive oil content. In a 2016 ALS mouse model study, De Paola et al. demonstrated that extra virgin oil extract acted as a neuroprotective agent in superoxide dismutase 1 (SOD1) mouse cultures by decreasing nitric oxide production from activated glia activated by the SOD1 mutation [13]. They reported that the toll-like receptor 4 (TLR4) signaling pathway, a pathogenic pathway in ALS, was downregulated by the olive oil extract. Moreover, olive oil contains hydroxytyrosol (HT), a polyphenol that shows anticancer, anti-inflammatory, neuroprotective and especially antioxidant properties [14]. In a different mouse model study, Oliván et al. found that dietary fat supplementation of extra virgin olive oil, mimicking the classical Mediterranean diet resulted in an amelioration of ALS pathological findings and delay in their onset in comparison to mice assigned to a stand mouse chow diet [15]. These animal studies suggest that the MedD can be implemented as an early intervention in ALS patients due to its antioxidant effects and neuroprotective ability. Interestingly, a clinical pilot study was done on Spanish participants in 2023 by Carrera-Julia et al. where they reported that Mediterranean Diet enriched with antioxidants nicotinamide riboside (NR) and pterostilbene (PTER), and the Mediterranean Diet combined with coconut oil (known as the ketogenic diet) are two different dietary approaches that can be implemented in ALS patients [16]. Their results show that participants who were assigned in the interventional groups GAx (Mediterranean diet with NR and PTER antioxidants), GCoco (Ketogenic Mediterranean diet and coconut oil) and the GAx + coco (Ketogenic Mediterranean diet, coconut oil, NR and PTER antioxidants) showed a significant improvement in anthropometric level when compared to those assigned in the control group. It is well known that NR and PTER supplementation can reduce oxidative stress, while coconut oil is an established substrate that can potentially alleviate mitochondrial dysfunction [17],[18]. Thus, polyphenol and antioxidants intake may be useful in reducing inflammation, as opposed to high fat or meat-based diets, which are associated with adverse health outcomes possibly due to the high content of proinflammatory foods and nutrients.

On the contrary, there have also been studies that have demonstrated that fruits, vegetables and antioxidants were shown to be associated with increased ALS function. In a 2016 study with over 302 ALS patients, Nieves et al. reported that higher intakes of antioxidants and carotenes from vegetables were associated with higher ALS Functional Rating Scale-Revised scores [19]. These findings suggested to re-evaluate incorporating fruit, vegetable and mediterranean diet in patients with ALS. Additionally, there are also studies that report antioxidants found in MedD such as vitamin E had no effect in patients with cognitive deficit or Alzheimer's disease [20]. Thus, further studies with effective methodological quality are necessary to warrant the effects of a Mediterranean diet rich in antioxidants in ALS patients.

### 2.2. Vegan Diet

A vegan diet has gained attention in the context of ALS for its potential role in both preventing the onset of the disease and slowing its progression. While ALS is a complex neurodegenerative disorder with multiple contributing factors that prove challenging to tackle, several key health benefits of a vegan diet make it a dietary choice of interest in the prevention and fight against this devastating condition. In a neuroepidemiology study conducted in Japan, the researchers examined the relationship between dietary habits. More specifically, they looked at the intake of vegetables, fruits and antioxidants alongside the risk of developing ALS [21]. Their findings indicated that individuals with a higher consumption of fruits and vegetables had a statistically significant reduction in the risk of ALS [21]. There was also a beneficial association between the intake of various types of vegetables and ALS risk. The study suggests that a diet rich in antioxidants found in fruits and vegetables may offer protection against the development of ALS [21].

The abundance of plant-based foods in a vegan diet, including fruits, vegetables, whole grains, nuts, seeds and legumes provides a rich source of antioxidants and anti-inflammatory compounds (Rahaman 2023). These components may help mitigate chronic neuroinflammation, which is believed to play a role in the development of ALS [22]. While an inflammatory reaction to neuronal injury is crucial for neuronal health and homeostasis, a chronic activation of the inflammatory response can potentially harm surrounding neurons and aggravate the disease process [23]. A meta-analysis included 21 cross-sectional studies and found that a vegan diet was associated with significantly lower levels of C-reactive protein (CRP) compared to omnivores [24]. Vegetarians also showed a modest reduction in CRP levels, although less pronounced. Notably, in individuals with impaired kidney function, the association between a vegetarian diet and CRP was more substantial [24]. However, no significant effects were observed for other inflammatory biomarkers studied. The study suggests a potential link between plant-based diets and reduced inflammation, particularly in the case of CRP, but underscores the need for further research, as most inflammatory biomarkers were only investigated in single studies. In another cross-sectional study comparing 36 vegans and 36 omnivores, researchers investigated the impact of a vegan diet on various inflammatory biomarkers. Surprisingly, no significant differences were found in inflammatory markers like high-sensitivity C-reactive protein (hsCRP), interleukin-18 (IL-18), interleukin-1 receptor antagonist (IL-1 RA), intercellular adhesion molecule 1 (ICAM-1), adiponectin, omentin-1 and resistin between the two groups (Menzel 2020). However, it was noted that the duration of a vegan diet was positively correlated with resistin, IL-18 and IL-1 RA levels [24]. The study also highlighted the influence of factors like body mass index (BMI) and waist circumference on the inflammatory state, emphasizing the need for further research to comprehensively understand how a vegan diet may affect the risk of chronic diseases through inflammatory mechanisms [24].

Excluding animal-based fats in a vegan diet is counterbalanced through the inclusion of plant-based healthy fats. Items such as avocados, flaxseeds and walnuts happen to be rich in omega-3 polyunsaturated fatty acids (PUFAS). Omega-3 PUFAS have been seen to be associated with neuroprotection and a decreased risk of ALS. This makes them a valuable piece of a vegan diet for those concerned with the prevention of ALS. A longitudinal study involving 1,002,082 participants (479,114 women; 522,968 men) from five prospective cohorts involved researchers examining the links between the dietary intake of omega-6 and omega-3 PUFAS and the risk of developing ALS. The study revealed that a higher intake of omega-3 PUFAS was associated with a reduced risk of developing ALS [25]. This was specifically seen in people with a larger consumption of α-linolenic and marine omega-3 PUFAS [25]. Contrarily, an intake of omega-6 PUFAS was not found to be associated with a risk of ALS at all. These findings suggest that including foods rich in omega-3 PUFAs in the diet may have a protective effect against ALS onset or progression [25]. In another case-control study involving 132 patients with ALS and 220 healthy controls, researchers assessed the relationship between premorbid dietary intake of various nutrients and the risk of developing ALS. The researchers found that a high intake of polyunsaturated fatty acids and vitamin E was significantly associated with a reduced risk of developing ALS [26]. Interestingly, when PUFA and vitamin E intake were analyzed together, they appeared to act synergistically, further reducing the risk of ALS [26].

### 2.3. Carnivore Diet

The carnivore diet, in contrast to the vegan diet, is based on consuming animal products while eliminating most or all plant-based foods. Diets containing high animal products have been associated with high amounts of saturated fat and low amounts of essential nutrients such as fiber among other nutritional deficiencies [27]. However, a recent 2020 study suggests that all essential nutrients can be obtained through a carnivore diet [28]. Carbohydrate content in animal products is minimal, thus the macromolecular composition of the carnivore diet resembles the ketogenic diet. Thus, as a result of low carbohydrate in the carnivore diet, ketone body production will be elevated. Ketone bodies have neuroprotective effects and have been demonstrated to have some level of neuroprotection in several neurological disorders such as epilepsy, Parkinson's and Alzheimer's. Through direct activation of G protein-coupled hydroxycarboxylic acid (HCA) receptors, particularly hydroxycarboxylic acid receptor 2 (HCA2), ketone bodies can elicit anti-inflammatory effects through inhibition of pro-inflammatory cytokines interleukin-1β (IL-1β) and IL-18. Additionally, ketone bodies decrease levels of glutamate and free radicals, thus providing neuroprotection at the mitochondrial level [29].

In the context of ALS, as the disease progresses and cognitive/behavioral changes develop, patients exhibit changes in eating behavior. Calorie intake, BMI and most notably, the consumption of saturated fat increases. Interestingly, Ahmed et al. found that these increased eating behavioral changes were associated with a threefold improved survival rate [30]. Patients with ALS typically under consume calories, consuming 82% of the daily recommended calorie intake [31]. Thus, the high caloric and high fat diet can be a compensatory measure to stabilize body weight. The role of fat foods in ALS is controversial as several studies have shown conflicting results. Some studies allude that a high-fat diet may slow or reduce the risk of ALS disease progression [4],[32]–[35]. However, other studies state that high-fat intake is correlated to a higher risk of ALS [5],[36],[37]. ALS pathogenesis involves increased oxidative stress, glutamate excitotoxicity, mitochondrial dysfunction. In mutant superoxide dismutase 1 mice models, high-fat and high calorie intake were shown to have improved mitochondrial function, survival rates and slowed disease progression [32].

Low-density lipoprotein (LDL) cholesterol is associated with ALS risk [36]. A 2021 study by Lennerz et al. analyzed the behavioral characteristics and self-reported health status of carnivore diet consumers and found a markedly elevated LDL [27]. A possible mechanism of LDL's neuroprotective effects is in axonal membrane assembly and growth. Cholesterol synthesis is reduced in peripheral nerve injuries. However, experimental models have shown an increase in LDL receptor expression to compensate for the reduced cholesterol synthesis and permitting the nerve to import cholesterol for axonal repair [38]. Thus, exogenous LDL from high fat diets can possibly increase the survival of peripheral motor neurons in ALS.

Pupillo et al. also found that there was an increased risk for ALS associated with total protein, animal protein and glutamate intake [5]. This is speculated to be due to the presence of glutamate in animal products, but the risk of dietary glutamate in the pathogenesis of ALS is unclear. High levels of glutamate can drive intracellular calcium influx and promote neuron death [39]. However, a recent study reported that higher intake of protein, especially meat, prolonged the survival rate of ALS patients [4].

### 2.4. Paleo Diet

A Paleolithic diet, otherwise known as paleo diet, consists of foods that humans may have eaten during the Paleolithic Era. Although there are different opinions on what this diet consists of, it usually incorporates small lean meats, seafood such as shellfish and salmon and plants such as nuts and seeds that could be acquired by hunters and gatherers based on their geographic location and the climate [40],[41]. A common theme, however, was that the paleo diet typically excludes foods such as dairy products, refined fats and sugar and processed foods that were discovered during the agricultural and industrial revolution [41],[42]. It was thought that changes in the human diet may have caused an increase in chronic diseases due to inadequacies in nutrition. This suggested that the paleo diet could play a role in mitigating the development of chronic diseases [42].

Within the context of ALS, the paleo diet may offer benefits through its emphasis on foods that may minimize inflammation. In normal individuals without ALS, the immune system often responds by activating the NF-κB pathway which mediates the production of pro-inflammatory cytokines [43]. However, a study testing the plasma of ALS patients showed abnormal levels of cytokines such as interleukin (IL)-1β, interleukin-2 (IL-2), interleukin-4 (IL-4), interleukin-5 (IL-5), interleukin-8 (IL-8), interleukin-10 (IL-10), interleukin-12p70 (IL-12p70), interleukin-13 (IL-13), tumor necrosis factor-α (TNF-α), interferon-γ (INF-γ), creatine kinase (CK) and ferritin compared to the control group [44]. Variable levels of these cytokines may play a role in the progression of the disease. One way the Paleo diet may alleviate symptoms of ALS is by promoting a nutrient-dense and anti-inflammatory dietary pattern. Although further research is needed to identify a direct connection between ALS and the paleo diet, studies have shown that for patients with other neurological diseases, such as multiple sclerosis, the paleo diet may interfere with the activation of the NF-κB pathway [6]. The diet allows for moderate amounts of polyunsaturated fats, mainly due to the higher proportion of omega-3 fatty acids [45]. Omega-3 fatty acids are involved with decreased activation of the NF-κB pathway, thereby inhibiting the expression of pro-inflammatory cytokines and preventing inflammation [7].

Findings from the study by Michels et al. revealed a link between high concentrations of total cholesterol and a heightened risk of developing ALS. Following a median observation period of 88.9 months and conducting a survival analysis with appropriate adjustments, they also discovered that increased levels of high-density lipoprotein (HDL) and LDL cholesterol were linked to elevated mortality rates among individuals with ALS [46]. The Paleolithic diet could potentially lower the likelihood of developing ALS, as studies have shown that it significantly decreases both total cholesterol and LDL cholesterol levels [47]. While the paleo diet's potential influence on inflammation and cholesterol levels may offer some insights to its relevance to ALS, further research is needed to establish any direct connections, highlighting the importance of ongoing research in the field of neurodegenerative diseases and diet.

### 2.5. Ketogenic Diet

The ketogenic diet, often referred to as the keto diet, describes a diet that is high in fat, moderate in protein and very low in carbohydrates. The purpose for this ratio of macronutrients is to stimulate the metabolic state of ketosis; in this state, drastically lowered levels of carbohydrates used as a fuel source of glucose induces the body to switch to using byproducts of fat metabolism in the liver as an alternative source of energy, known as ketones [48]. Ketone production can be sustained with continuous carbohydrate deprivation and fat breakdown, and ketones can be utilized by vital organs, including the brain by crossing the blood-brain barrier, without significantly altering blood pH [49].

Ketogenic diets differ from “low-carbohydrate diets” in that ketogenic diets typically limit daily carbohydrate intake to less than 50 grams, while low-carbohydrate diets can allow up to approximately 130 grams per day [48]. The macronutrient composition of a general ketogenic diet ranges from around 55–60% of energy received from fats, 30–35% from proteins and 5–10% from carbohydrates, and these percentages can be modified based on individual preference. However, a restricted carbohydrate diet is not enough to induce ketosis, as an increased fat and protein intake is needed to be utilized as a primary fuel source to replace carbohydrates. The primary goal of the ketogenic diet is to target elevated body fats as a rapid and effective approach to weight loss, as well as an indication for use in the treatment of chronic disease [49].

Ketogenic diets have been found to be an effective therapeutic intervention in several neurodegenerative diseases, including medication-resistant epilepsy, Alzheimer's disease and Parkinson's disease [8]. One proposed mechanism for the neurodegeneration seen in ALS is mitochondrial dysfunction. A study utilizing human induced pluripotent stem cells compared to generated experimental familial ALS models provides a strong link between mitochondrial damage and the oxidative stress and DNA damage seen in ALS neuropathy [50]. Ketone metabolism has been shown to increase production of essential citric acid cycle substrates such as acetyl coenzyme A (acetyl-CoA) and decrease mitochondrial free radical generation and thus ketogenic diets may offer neuroprotection and potentially slow progression of damage for the motor neurons involved in the ALS disease process [51]. The neuroprotective effects of the ketogenic diet have been supported by experimental mouse models of multiple sclerosis, reporting symptomatic improvement of pathology through elevated expression of myelin basic protein and mature oligodendrocytes and reduced demyelination of hippocampal neurons. Other murine studies point to ketones' correlation with decreased expression of the pro-inflammatory molecules NF-κB and TNFα, further supporting the neuroprotective effect of the ketogenic diet against inflammation [52]. Much like the carnivore diet, which is similarly high in fat and low in carbohydrates, studies suggest that an increased dietary intake of fat may reduce the risk of ALS and slow the rate of the condition's progression in association with ketone neuroprotection against oxidative stress [32],[35]. However, opposing results may be seen in a controlled study that suggests a high caloric diet with increased carbohydrate intake correlated to greater survival and positive outcomes in comparison to a high fat diet [34]. Further studies find that a high fat diet may even increase the oxidative stress possibly involved in ALS pathogenesis and may increase the risk of sporadic ALS [36],[37]. Therefore, a dietary approach to ALS that is high in fat and low in carbohydrates may be shown to prevent development of the condition and slow further disease progression.

## 3. Conclusion

ALS is a multi-faceted and complex disease, of which the specific causes that lead to the disease are still unknown. However, recent studies have shown that diet can be an important factor that can reduce the risk of neurodegenerative diseases such as ALS. These diets have been shown to have a positive effect on several other neurological disorders such as Alzheimer's, Parkinson's and dementia. This review highlights several popular dietary patterns and the potential effects they may have on the onset or progression of ALS. Although these diets vary in macromolecule composition and food intake, the neuroprotective effects generally involve the intake of anti-inflammatory and antioxidant nutrients to reduce the oxidative stress of ALS. However, it was noted that the potential benefits of each diet and their composition conflicted in the different studies that were analyzed. Many variables can influence the results of these diets such as genetic susceptibility, environment and other lifestyle aspects as well as the interaction of these different variables. Thus, a greater understanding of the progression and risk factors for ALS can allow patients to better tailor their dietary intake. Elucidating the beneficial or harmful mechanisms of certain foods can provide a greater understanding of the pathogenesis of this disease. The limitations of these studies are the heterogeneity of the studies included and the type of studies included. There is no current standardized approach to evaluating dietary impact and controlling for the many variables that can influence the results of each study. Due to the conflicting views of the benefits of these diets in the literature, future studies should conduct randomized controlled trials (RCTs) with standardized methodologies and patient populations to more effectively evaluate the effects of the diets discussed in this study.

## References

[1] Masrori P, Van Damme P (2020). Amyotrophic lateral sclerosis: a clinical review. Eur J Neurol.

[2] Caplliure-Llopis J, Peralta-Chamba T, Carrera-Juliá S (2020). Therapeutic alternative of the ketogenic Mediterranean diet to improve mitochondrial activity in Amyotrophic Lateral Sclerosis (ALS): A Comprehensive Review. Food Sci Nutr.

[3] Yip PK, Pizzasegola C, Gladman S (2013). The omega-3 fatty acid eicosapentaenoic acid accelerates disease progression in a model of amyotrophic lateral sclerosis. PLoS One.

[4] Kim B, Jin Y, Kim SH (2020). Association between macronutrient intake and amyotrophic lateral sclerosis prognosis. Nutr Neurosci.

[5] Pupillo E, Bianchi E, Chiò A (2018). Amyotrophic lateral sclerosis and food intake. Amyotroph Lat Scl Fr.

[6] Irish A, Erickson C, Wahls T (2017). Randomized control trial evaluation of a modified Paleolithic dietary intervention in the treatment of relapsing-remitting multiple sclerosis: a pilot study. Degener Neurol Neuromuscul Dis.

[7] Djuricic I, Calder PC (2021). Beneficial Outcomes of Omega-6 and Omega-3 Polyunsaturated Fatty Acids on Human Health: An Update for 2021. Nutrients.

[8] Paoli A, Bianco A, Damiani E (2014). Ketogenic Diet in Neuromuscular and Neurodegenerative Diseases. BioMed Res Int.

[9] Altomare R, Cacciabaudo F, Damiano G (2013). The mediterranean diet: a history of health. Iran J Public Health.

[10] Andreu-Reinón ME, Chirlaque MD, Gavrila D (2021). Mediterranean Diet and Risk of Dementia and Alzheimer's Disease in the EPIC-Spain Dementia Cohort Study. Nutrients.

[11] Gantenbein KV, Kanaka-Gantenbein C (2021). Mediterranean Diet as an Antioxidant: The Impact on Metabolic Health and Overall Wellbeing. Nutrients.

[12] Shen N, Wang T, Gan Q (2022). Plant flavonoids: Classification, distribution, biosynthesis, and antioxidant activity. Food Chem.

[13] De Paola M, Sestito SE, Mariani A (2016). Synthetic and natural small molecule TLR4 antagonists inhibit motoneuron death in cultures from ALS mouse model. Pharmacol Res.

[14] Hu T, He XW, Jiang JG (2014). Hydroxytyrosol and its potential therapeutic effects. J Agric Food Chem.

[15] Oliván S, Martínez-Beamonte R, Calvo AC (2014). Extra virgin olive oil intake delays the development of amyotrophic lateral sclerosis associated with reduced reticulum stress and autophagy in muscle of SOD1G93A mice. J Nutr Biochem.

[16] Carrera-Juliá S, Estrela JM, Zacarés M (2023). Effect of the Mediterranean diet supplemented with nicotinamide riboside and pterostilbene and/or coconut oil on anthropometric variables in amyotrophic lateral sclerosis. A pilot study. Front Nutr.

[17] Dellinger RW, Santos SR, Morris M (2017). Repeat dose NRPT (nicotinamide riboside and pterostilbene) increases NAD+ levels in humans safely and sustainably: a randomized, double-blind, placebo-controlled study. NPJ Aging Mech Dis.

[18] Napolitano G, Fasciolo G, Di Meo S (2019). Vitamin E Supplementation and Mitochondria in Experimental and Functional Hyperthyroidism: A Mini-Review. Nutrients.

[19] Nieves JW, Gennings C, Factor-Litvak P (2016). Association Between Dietary Intake and Function in Amyotrophic Lateral Sclerosis. JAMA Neurol.

[20] Desnuelle C, Dib M, Garrel C (2001). A double-blind, placebo-controlled randomized clinical trial of α-tocopherol (vitamin E) in the treatment of amyotrophic lateral sclerosis. Amyotroph Lat Scl Motor Neuron Disord.

[21] Okamoto K, Kihira T, Kobashi G (2009). Fruit and vegetable intake and risk of amyotrophic lateral sclerosis in Japan. Neuroepidemiology.

[22] Rahaman MM, Hossain R, Herrera-Bravo J (2023). Natural antioxidants from some fruits, seeds, foods, natural products, and associated health benefits: An update. Food Sci Nutr.

[23] Masrori P, Beckers J, Gossye H (2022). The role of inflammation in neurodegeneration: novel insights into the role of the immune system in C9orf72 HRE-mediated ALS/FTD. Mol Neurodegener.

[24] Menzel J, Biemann R, Longree A (2020). Associations of a vegan diet with inflammatory biomarkers. Sci Rep.

[25] Fitzgerald KC, O'Reilly ÉJ, Falcone GJ (2014). Dietary ω-3 polyunsaturated fatty acid intake and risk for amyotrophic lateral sclerosis. JAMA Neurol.

[26] Veldink JH, Kalmijn S, Groeneveld GJ (2007). Intake of polyunsaturated fatty acids and vitamin E reduces the risk of developing amyotrophic lateral sclerosis. J Neurol Neurosurg Psychiatry.

[27] Lennerz BS, Mey JT, Henn OH (2021). Behavioral characteristics and self-reported health status among 2029 adults consuming a “carnivore diet.”. Curr Dev Nutr.

[28] O'Hearn A (2020). Can a carnivore diet provide all essential nutrients?. Curr Opin Endocrinol Diabetes Obes.

[29] Caplliure-Llopis J, Peralta-Chamba T, Carrera-Juliá S (2020). Therapeutic alternative of the ketogenic Mediterranean diet to improve mitochondrial activity in Amyotrophic Lateral Sclerosis (ALS): A Comprehensive Review. Food Sci Nutr.

[30] Ahmed RM, Caga J, Devenney E (2016). Cognition and eating behavior in amyotrophic lateral sclerosis: effect on survival. J Neurol.

[31] Kasarskis EJ, Berryman S, Vanderleest JG (1996). Nutritional status of patients with amyotrophic lateral sclerosis: relation to the proximity of death. Am J Clin Nutr.

[32] Paganoni S, Wills AM (2013). High-fat and ketogenic diets in amyotrophic lateral sclerosis. J Child Neurol.

[33] Okamoto K, Kihira T, Kondo T (2007). Nutritional status and risk of amyotrophic lateral sclerosis in Japan. Amyotroph Lateral Scler.

[34] Wills AM, Hubbard J, Macklin EA (2014). Hypercaloric enteral nutrition in patients with amyotrophic lateral sclerosis: a randomised, double-blind, placebo-controlled phase 2 trial. Lancet.

[35] Yang LP, Fan DS (2017). Diets for patients with amyotrophic lateral sclerosis: Pay attention to nutritional intervention. Chin Med J (Engl).

[36] D'Antona S, Caramenti M, Porro D (2021). Amyotrophic lateral sclerosis: A diet review. Foods.

[37] Huisman MHB, Seelen M, van Doormaal PTC (2015). Effect of presymptomatic body mass index and consumption of fat and alcohol on amyotrophic lateral sclerosis. JAMA Neurol.

[38] Vance JE, Campenot RB, Vance DE (2000). The synthesis and transport of lipids for axonal growth and nerve regeneration. Biochim Biophys Acta Mol Cell Biol Lipids.

[39] Iwasaki Y, Ikeda K, Kinoshita M (2002). Molecular and cellular mechanism of glutamate receptors in relation to amyotrophic lateral sclerosis. Curr Drug Targets CNS Neurol Disord.

[40] Frączek B, Pięta A, Burda A (2021). Paleolithic Diet-Effect on the Health Status and Performance of Athletes?. Nutrients.

[41] de Menezes EVA, Sampaio HA de C, Carioca AAF (2019). Influence of Paleolithic diet on anthropometric markers in chronic diseases: systematic review and meta-analysis. Nutr J.

[42] Singh A, Singh D (2023). The Paleolithic Diet. Cureus.

[43] Liu T, Zhang L, Joo D (2017). NF-κB signaling in inflammation. Sig Transduct Target Ther.

[44] McCauley ME, Baloh RH (2019). Inflammation in ALS/FTD pathogenesis. Acta Neuropathol.

[45] Tarantino G, Citro V, Finelli C (2015). Hype or Reality: Should Patients with Metabolic Syndrome Related NAFLD be on the Hunter-Gatherer (Paleo) Diet to Decrease Morbidity?. JGLD.

[46] Michels S, Kurz D, Rosenbohm A (2023). Association of blood lipids with onset and prognosis of amyotrophic lateral sclerosis: results from the ALS Swabia registry. J Neurol.

[47] Frassetto LA, Schloetter M, Mietus-Synder M (2009). Metabolic and physiologic improvements from consuming a paleolithic, hunter-gatherer type diet. Eur J Clin Nutr.

[48] Crosby L, Davis B, Joshi S (2021). Ketogenic Diets and Chronic Disease: Weighing the Benefits Against the Risks. Front Nutr.

[49] Masood W, Annamaraju P, Khan Suheb MZ (2023). Ketogenic Diet.

[50] Singh T, Jiao Y, Ferrando LM (2021). Neuronal mitochondrial dysfunction in sporadic amyotrophic lateral sclerosis is developmentally regulated. Sci Rep.

[51] Veech RL (2004). The therapeutic implications of ketone bodies: the effects of ketone bodies in pathological conditions: ketosis, ketogenic diet, redox states, insulin resistance, and mitochondrial metabolism. Prostaglandins Leukot Essent Fatty Acids.

[52] Gough SM, Casella A, Ortega KJ (2021). Neuroprotection by the ketogenic diet: Evidence and controversies. Front Nutr.

